# Mastering your Fellowship: Part 2, 2025

**DOI:** 10.4102/safp.v67i1.6069

**Published:** 2025-03-04

**Authors:** Mergan Naidoo, Klaus von Pressentin, John M. Musonda, Selvandran Rangiah, Chantelle van der Bijl

**Affiliations:** 1 Discipline of Family Medicine, College of Health Sciences, University of KwaZulu-Natal, Durban, South Africa; 2 Department of Family, Community and Emergency Care, Faculty of Health Sciences, University of Cape Town, Cape Town, South Africa; 3 Department of Family Medicine and Primary Care, Faculty of Health Sciences, University of the Witwatersrand, Johannesburg, South Africa; 4 Department of Family Medicine, Faculty of Health Sciences, University of the Free State, Durban, South Africa

**Keywords:** family physicians, FCFP (SA) examination, family medicine registrars, postgraduate training, national exit examination, peripartum care

## Abstract

The ‘Mastering your Fellowship’ series provides examples of the question format encountered in the written and clinical examinations for the Fellowship of the College of Family Physicians of South Africa (FCFP [SA]) examination. The series is aimed at helping family medicine registrars prepare for this examination.

## Introduction

This section in the *South African Family Practice* journal is aimed at helping registrars prepare for the Fellowship of the College of Family Physicians of South Africa (FCFP [SA]) examination. It will provide examples of the question formats encountered in the written examination: a multiple choice question (MCQ) in the form of a single best answer (SBA – Type A) or extended matching question (EMQ – Type R); short answer questions (SAQ), questions based on the critical reading of a journal (evidence-based medicine) and an example of an objectively structured clinical examination (OSCE) question. Each question type is presented based on the College of Family Physicians blueprint and the key learning outcomes of the FCFP programme. The MCQs are based on the 10 clinical domains of family medicine, and the SAQs will be aligned with the five national unit standards. The critical reading section will include evidence-based medicine and primary care research methods.

This article is based on entrustable professional activity (EPA) 1 (Managing women and newborns in the peripartum period), and EPA 22 (Leading clinical governance activities). We suggest you attempt to answer the questions (by yourself or with peers or supervisors) before finding the model answers online: http://www.safpj.co.za/.

Please visit the Colleges of Medicine website for guidelines on the Fellowship examination: https://cmsa.co.za/fellowship-of-the-college-of-family-physicians-of-south-africa-fcfpsa/.

We are keen to hear about how this series assists registrars and their supervisors in preparing for the FCFP (SA) examination. Please email us (editor@safpj.co.za) your feedback and suggestions.

## Multiple choice question (MCQ): Single best answer

A 30-year-old female, parity 1, gravida 2 (P1G2), at 29 weeks gestation, presents to your district hospital with a sudden gush of fluid from her vagina about 4 h ago. She is not in pain. Her antenatal clinic visits started early, and she attended three times with unremarkable antenatal care (ANC) blood results. She spontaneously delivered at 36 weeks in her previous pregnancy. Her vitals are: blood pressure (BP) = 128/82 mmHg, pulse (P) = 90 beats/min, respiratory rate (RR) = 18 breaths/min, Temperature = 37.3 °C. Which is the most appropriate next step in her management?

- Do a digital vaginal examination.
- Assess the foetal wellbeing.
- Determine the pH of vaginal fluid.
- Do a speculum vaginal inspection.
- Do an ultrasound examination to assess the liquor.

Short answer: (d)

Prematurity is one of the most common causes of perinatal mortality and morbidity. Infants born before 37 weeks gestation are preterm. Most problems occur before 34 weeks gestation, when neonatal issues are at their highest. Premature Prelabour Rupture of Membranes (PPROM) is a rupture of the membranes before the onset of labour and 37 weeks of gestation. Confirmation of the diagnosis is by a visual inspection by a sterile vagina speculum examination, pH testing of vaginal fluid or, if necessary, liquor volume assessment on ultrasound. Avoid the digital vaginal examination. Preterm labour (PTL) occurs after the gestation of ≥ 24 weeks and before 37 completed weeks of pregnancy. It is confirmed by regular uterine contractions (at least once every 10 min), cervical changes and rupture of the membrane.

Some general measures in the management of both PTL and PPROM include assessment of foetal wellbeing, estimation of foetal weight by palpation or ultrasound and a high index of suspicion of chorioamnionitis. Delivery of the baby is recommended if chorioamnionitis is suspected. A previous spontaneous preterm delivery is a high-risk factor for preterm delivery in the subsequent pregnancy. The careful consideration of either cervical cerclage or vaginal progesterone therapy may prolong pregnancy in some instances.

Suppressing regular uterine contractions depends on the gestation age at presentation. If it is less than 34 weeks, pre-hydrate with sodium chloride 0.9%, intravenous (IV), 200 mL, before administration of Nifedipine oral, 20 mg. Follow it up with 10 mg after 30 min, then 10 mg 4 hourly for up to 48 h if uterine contractions persist. If gestation is less than 32 weeks, and Nifedipine is contra-indicated, like in chorioamnionitis, indomethacin, oral, 50 mg, then 25 mg 4 hourly for up to 48 h.

To improve foetal lung maturity at 26–34 weeks, administer betamethasone 12 mg intramuscularly (IM) twice or dexathamesone 8 mg three times a day, soon after diagnosis of PTL or PPROM is made. Further, administer antibiotics ampicillin, IV, 1 g 6 hourly for 48 h, followed by Amoxicillin, oral, 500 mg 8 hourly for 5 days. Also, give 1g of azithromycin orally as a single dose if the patient is not allergic to penicillin. Finally, prepare for appropriate care of the preterm baby and referral if needed.

**Further reading**

- National Department of Health. South African adult hospital healthcare level essential medicines list: Chapter 6: Obstetrics: NEMLC Recommendations for Medicine Amendments 2020–3. Pretoria: National Department of Health of South Africa; 2024.
- National Department of Health. Guidelines for maternity care in South Africa – A manual for clinics, community health centres and district hospitals. 4th ed. Pretoria: National Department of Health of South Africa; 2016.

## Short answer question (SAQ): Entrustable professional activity 22: Leading clinical governance activities

You are a family physician based at an urban community health centre. A recent survey from your sub-district on gender-based violence (GBV) shows that there is an increase in reported sexual (8% ever experienced) and physical violence (25% ever experienced) in women 18 years and older, compared to the national averages (6.2% and 20.5%, respectively). Answer the following questions related to this scenario.

1. **Your primary health care (PHC) management team are reviewing the existing relationships with other sectors that share the task of addressing this issue of GBV experienced by your community. Name a few important sectors and government departments with which to build relationships. (5 marks)**

Any 5 of the following:

- Department of Social Development.
- Department of Police and Community Safety.
- Department of Women, Youth and Persons with Disabilities.
- Department of Basic Education.
- Local Government and municipality (metropolitan, district and local municipalities).
- Social Protection and Community Development Cluster.
- Non-governmental organisations (NGOs).

2. **The team wants to work more collaboratively with the other sectors. At the first intersectoral team meeting, you are asked to facilitate a session to build consensus on possible interventions to address the issue of GBV in your community. Give examples of how you would lead the session to ensure that collaboration is built and strengthened. (5 marks)**

Any 5 of the following:

- Respect: Ensure that when you facilitate, you build the notion of mutual respect. For introductions, use a round table (circle) and ice-breakers.
- Joint knowledge creation: Ensure that all present are equally involved in the meeting and give input. Focus on the participants who do not give their opinion and specifically ask them.
- Listening, open communication and feedback: As a facilitator, try not to give your opinion (or at least after everybody has spoken). Use reflection and summaries during the session, affirming when a person gives an opinion.
- Equal power relationships: Make sure one group is not overrepresented. This means from each group, there are similar representations in the meeting.
- Building network(s) and structure: Ensure follow-up meetings and establish memorandums of understanding (MOUs).
- Equal effort: Make use of consensus for conclusions. Round robin, voting.
- Judgement and blame: Defuse any blaming or judgement in the meeting.
- Set goals collaboratively.

3. **Your PHC team met to make a plan for how they can contribute to the fight against GBV. Discuss a few practical options for when the ward-based outreach team (WBOT) can be involved in screening, prevention and promotion activities. (5 marks)**

Any 5 marks related to the scenario:

- Create formal linkages and partnerships with NGOs such as Women on Farms, People Opposing Women Abuse (POWA), Rape Crisis, Masimanyane Women’s Rights International, Rural Development Support Program and TEARS (Transform Education About Rape and Sexual abuse) Foundation. These NGOs facilitate shelter services for clients (and their children where relevant).
- Raise awareness about GBV and available services to help during home visits.
- Screening for GBV during home visits, including maternal health users.
- Support groups for GBV linked to local government and NGOs.
- Community education about GBV (raise awareness) – flyers, community radio and newspaper, door-to-door, community forums.
- Community meetings with leaders from faith organisations, local community safety and health activists.

4. **The management team ask you, as the family physician, to work with the hospital and PHC on a plan to fight against GBV. Make a few suggestions on how clinicians in the hospital and PHC can contribute to the fight against GBV and suggest a process to implement it. (5 marks)**

Suggestions:

- Improved screening: In-service training to raise awareness.
- Develop a screening tool to implement during consultations.
- Information session at the clinic waiting area.
- Use the quality improvement cycle or Plan-Do-Study-Act (PDSA) cycle for quality improvement as the process.

Plan an intervention together (co-creation), implement/do, study/evaluate, act.

For example, plan and adapt a screening intervention to be used in the Outpatient department (OPD) or antenatal clinic, which may be linked to information and educational sessions. Create a care pathway for those who screen positive for GBV and ensure that there is a person-centred mechanism to connect the victim with social services and mental health support, including support groups and NGOs. Share statistics and anonymised experiences with a multi-sectoral working group that is mandated to monitor and evaluate the intervention.

5. **Two interns were threatened in the emergency room by the partner of a GBV patient. They approach you and confide that they are at risk of burnout. Describe key elements of building resilience, which you will share in a private space during a mentoring conversation. (5 marks)**

Enabling resilience: (any 5 of these ‘P’ activities)

- People: Sharing problems, thoughts and feelings with others (partners, peers, practitioners and professionals – Balint groups).
- Physical care: Sleep, diet, activity, relaxation.
- Pursuits outside of medicine: Hobby, sports, cultural interests.
- Private reflection: Journal, meditating, mindfulness.
- Purpose: Defining your direction and setting and seeking goals.
- Prayer: Developing spiritual dimensions.

**25 marks**

**Further reading**

- The status of women’s health in South Africa: Evidence from selected indicators / Statistics South Africa [homepage on the Internet]. Pretoria: Statistics South Africa; 2022 [cited 2024 Oct 01]. Available from: http://www.statssa.gov.za/publications/03-00-18/03-00-182022.pdf
- Mash R, Gaede B, Hugo JJ. The contribution of family physicians and primary care doctors to community-orientated primary care. S Afr Fam Pract. 2021;63(1):e1–e5. https://doi.org/10.4102/safp.v63i1.5281
- Hugo J. Chapter 18: How to link the PHC teams to the rest of the health system, other sectors and the community. In: Mash B, Brits H, Naidoo M, Ras T, editors. South African Family Practice Manual. 4th ed. Pretoria: Van Schaik Publishers, 2023; p. 65–70.
- Jenkins L. Chapter 190: How to take on leadership roles and enable teamwork. In: Mash B, Brits H, Naidoo M, Ras T, editors. South African Family Practice Manual. 4th ed. Pretoria: Van Schaik Publishers, 2023; p. 766–773.
- Viljoen W. Chapter 191: How to organise and run M&M meetings. In: Mash B, Brits H, Naidoo M, Ras T, editors. South African Family Practice Manual. 4th ed. Pretoria: Van Schaik Publishers, 2023; p. 774–775.
- Wagner L. Chapter 194: How to enable resilience and avoid burnout. In: Mash B, Brits H, Naidoo M, Ras T, editors. South African Family Practice Manual. 4th ed. Pretoria: Van Schaik Publishers, 2023; p. 781–783.

## Critical appraisal of research

Read the accompanying article carefully and then answer the following questions. As far as possible, use your own words. Do not copy out chunks from the article. Be guided by the allocation of marks concerning the length of your responses.

- Stofberg JP, Spittal GW, Hinkel T, Ras T. A descriptive study of suspected perinatal asphyxia at Mitchells Plain District Hospital: A case series. S Afr Fam Pract. 2020;62(4):a5112. https://doi.org/10.4102/safp.v62i1.5112

**Total: 30 marks**

1. Identify three distinct arguments made by the authors to justify and provide a rationale for the study. (3 marks)
2. Critically appraise the authors’ choice of study design to answer the research question and limit bias. (6 marks)
3. Critically appraise the description of the study population. (3 marks)
4. Describe and critically appraise how the authors went about with ‘case finding’ for this case series. (7 marks)
5. Critically appraise the measurement of the outcomes of interest. (3 marks)
6. Critically appraise the approach used by the authors to validate their data collection tool regarding content, construct and face validation. (6 marks)
7. Critically appraise the appropriateness of the statistical analysis. (2 marks)

**Suggested answers**

**Identify three distinct arguments made by the authors to justify and provide a rationale for the study. (3 marks)**
  - High neonatal mortality rates, with perinatal asphyxia being a leading cause, justify investigating contributing factors, especially in South Africa, where it is difficult to estimate the prevalence of this condition.
  - There is a lack of research on perinatal asphyxia in district-level hospitals like Mitchells Plain, a study setting where over 50% of all perinatal asphyxia-related deaths occur.
  - Identifying modifiable factors in peripartum and postpartum care could improve neonatal outcomes in district-level care settings, especially given the time-dependant intricate decision-making in diagnosing and managing neonates who suffered perinatal asphyxia with suspected hypoxic ischaemic encephalopathy (HIE).
**Critically appraise the authors’ choice of study design to answer the research question and limit bias. (6 marks)**
  - The authors aimed to describe the clinical care of the mother and baby dyad at a district hospital to identify possible preventable contributors to perinatal asphyxia.
  - The retrospective descriptive case series is appropriate for identifying patterns and associations in perinatal asphyxia.
  - However, this retrospective design introduces bias, as it relies on records that may be incomplete or inaccurate. This limits the ability to establish causal relationships between modifiable factors and outcomes.
  - The case series design chosen in this study has a potential selection bias, a concern, as the study only included referred cases and excluded milder cases.
  - No use of multivariate analysis in the analytical component to adjust for confounders, weakening the ability to assess independent effects.
  - A prospective cohort design or a matched control study would address confounding and causal inference better.
**Critically appraise the description of the study population. (3 marks)**
  - The population was well-defined, focussing on neonates referred from Mitchells Plain District Hospital (MPH) to Groote Schuur (GSH) and Mowbray Maternity Hospitals (MMH) with suspected perinatal asphyxia.
  - Clear inclusion criteria were defined, such as Apgar ≤ 7 at 5 min and clinical signs of moderate to severe encephalopathy, which are appropriate for suspected perinatal asphyxia cases. Babies also had the following factors: gestation of 36 weeks or more, birthweight of 1.8 kg or more and referred from MPH to the two hospitals.
  - Clear exclusion criteria (e.g. comorbidities, congenital anomalies) that could influence neonatal outcomes were described.
**Describe and critically appraise how the authors went about with ‘case finding’ for this case series. (7 marks)**
  - The authors identified cases of suspected perinatal asphyxia referred between 2016 and 2018.
  - The case finding was performed by reviewing electronic medical records from the hospital’s content management system extracellular matrix electronic case management (ECM) of all neonates transferred from the neonatal unit during the study period.
  - A total of 33 cases were initially identified, and after applying the study’s inclusion and exclusion criteria, 29 cases were included in the final analysis.
  - The strengths of the case-finding approach include: (any 2 points).
    ■ Comprehensive coverage: By reviewing all neonatal transfers over a multi-year period (2016–2018), the authors ensured that a wide range of potential cases were captured, increasing the likelihood that all relevant cases of suspected perinatal asphyxia were included.
    ■ Electronic records: Using an ECM for case identification is efficient and likely improves the accuracy of case finding by reducing the risk of missing records that might occur with paper-based systems.
    ■ Inclusion and exclusion criteria: The criteria for selecting cases (such as Apgar scores, need for resuscitation, or signs of HIE) were clinically appropriate and consistent with the study’s aim to focus on suspected cases of perinatal asphyxia. Excluding neonates with significant comorbidities like congenital anomalies was justified to avoid confounding variables.
  - The weaknesses of the case-finding approach include: (any 2 points)
    ■ Retrospective nature: Retrospective case finding is inherently limited by the accuracy and completeness of medical records. Important data could be missing, inaccurately recorded, or inconsistently documented, potentially leading to selection or information bias.
    ■ Single-hospital focus: The case finding was limited to a single district hospital, limiting the findings’ generalisability. Cases from other district hospitals or those managed at lower levels of care (e.g. midwife obstetric units) were excluded, meaning the study may not represent the full spectrum of perinatal asphyxia in the service drainage area.
    ■ Exclusion of mild cases: The study primarily focussed on severe cases referred to tertiary centres. Neonates with milder forms of perinatal asphyxia who were not referred might have been missed, potentially introducing selection bias by skewing the sample towards more severe cases.
    ■ Potential for missed cases: Neonates with perinatal asphyxia who died before transfer or who were not referred due to the perceived mildness of the condition may not have been captured, leading to underrepresentation of early neonatal deaths or less severe cases of perinatal asphyxia.
**Critically appraise the measurement of the outcomes of interest. (3 marks)**
  - The outcomes of interest were not clearly described in the data analysis section of the methods section.
  - However, Tables 5a and 5b in the results section show the primary outcomes as normal or abnormal amplitude-electroencephalogram (aEEG) and mortality (alive or demised at day 7 of life).
  - The gold-standard diagnostic special investigation, an aEEG, is valid and clinically relevant for assessing perinatal asphyxia outcomes. Retrospective reliance on clinical records may usually introduce inconsistencies in measuring outcomes. However, the aEEG and mortality at day seven are objective measures.
6. **Critically appraise the approach used by the authors to validate their data collection tool regarding content, face and construct validation. (6 marks)**
  - Content validation refers to ensuring that a data collection tool adequately covers all aspects of the measured concept.
    ■ The authors state that the data collection tool was based on existing literature, which is an appropriate approach to ensure the tool encompasses relevant clinical variables (e.g., clinical signs of perinatal asphyxia, Apgar scores, etc.).
  - Face validation refers to the extent to which a data collection tool appears to measure what it is supposed to measure based on a superficial assessment. It focusses on the tool’s appropriateness, clarity and relevance as perceived by individuals who will use it, such as researchers or clinicians. While face validity is less rigorous than content validity, it ensures the tool seems reasonable and understandable for its intended purpose.
    ■ The tool was reviewed by key role players at the district hospital (Mitchells Plain) and specialist neonatologists and obstetricians, indicating that subject matter experts were involved in the validation process.
  - Construct validation assesses how well a test measures the concept it intended to measure. It is the degree to which performance on a measure represents the level of ability or degree of the measured construct and usually involves factor analysis and data reduction.
    ■ While the tool was pilot-tested with 10 cases, the article does not specify whether feedback from the pilot users (clinicians or data collectors) was systematically collected and analysed, which would have provided further insights into the tool’s effectiveness and usability.
7. **Critically appraise the appropriateness of the statistical analysis. (2 marks)**
  - Descriptive statistics and chi-square tests are appropriate for examining categorical variables and their associations.
  - Lack of advanced statistical analysis (e.g. logistic regression) means the study does not adjust for confounding variables, limiting the strength of the findings.

**Further reading**

- Pather M. Evidence-based family medicine. In: Mash B, editor. Handbook of family medicine. 4th ed. Cape Town: Oxford University Press, 2017; p. 430–453.
- The Critical Appraisals Skills Programme (CASP). CASP checklists [homepage on the Internet]. 2024 [cited 2024 Oct 07]. Available from: https://casp-uk.net/casp-tools-checklists/

## Objectively structured clinical examination station (OSCE): Entrustable professional activity 1: Managing women and newborns in the peripartum period

*Objective of station*: This station tests the candidate’s ability to teach a medical intern how to explain and demonstrate Kangaroo Father Care (KFC) to a father.

*Type of station*: Integrated consultation.

*Role players*: A medical intern who is unfamiliar with KFC and is seeking guidance on how to teach it to the father of a newborn.

Instructions for candidate:

- Mr Sam, a father in the paediatric ward, asks the intern to teach him how to do KFC, because the mother will go home for a while during the day.
- They inform the intern that they have only heard of it before and have never done it practically.
- The intern has not heard about Kangaroo Mother Care (KMC) nor KFC.
- Explain to the intern more about KMC and KFC.
- Show the intern how to position the baby and father for KFC.

### Instructions for the examiner

- This is an integrated consultation station in which the candidate has 20 min.
- Familiarise yourself with the assessor guidelines, which detail the expected responses from the candidate (Table 1).
- No marks are allocated. In the mark sheet, tick off one of the three responses for each competency listed. Ensure you are clear on the criteria for judging a candidate’s competence in each area.

**TABLE 1:** Marking sheet for consultation station.

Competencies	Candidate’s rating		
	Not competent	Competent	Good
Establishes and maintains a good clinician-patient relationship and supervisor-intern relationship.	-	-	-
Gathering information: History/examination/investigations	-	-	-
Clinical reasoning	-	-	-
Explanation and Planning to the patient	-	-	-
Management	-	-	-

### Guidance for examiners

- The aim is to establish that the candidate has an effective and safe approach to managing KMC and KFC by demonstrating their ability to teach a junior colleague about the concept and its implementation.
- A working definition of competent performance is when the candidate effectively completes the task within the allotted time in a manner that maintains patient safety, even though the execution may not be efficient and well structured.
  ■ *Not competent*: patient safety is compromised (including ethically and legally), or the task is not completed.
  ■ *Competent*: the task is completed safely and effectively.
  ■ *Good*: in addition to displaying competence, the task is completed efficiently and in an empathic, patient-centred manner (acknowledges patient’s ideas, beliefs, expectations, concerns/fears).

### Establishes and maintains a good doctor-patient relationship

The competent candidate is respectful and engages with the intern in a dignified manner. (*Ascertains reason for the consultation and makes the intern feel comfortable while ensuring the ground for confidentiality is set.*) The good candidate is empathic, compassionate and collaborative, facilitating active participation in key areas of the consultation. (*Maintains this throughout the consultation.*)

### Gathering information

The competent candidate gathers sufficient information to establish a clinical assessment. (*This includes establishing baseline knowledge of the intern as well as what the intern wants to learn; identifying readiness and any concerns about performing the care, ensuring that the infant is stable enough for KFC, the intern can ascertain the duration the father will be responsible for KFC, and assessing any support systems in place. Discusses issues of teaching methods and assessment.*)

The good candidate additionally has a structured and holistic approach. (*Ascertains if the mother has fully recovered from any childbirth complications and the reason for the involvement of the father; identify any psychological concerns the father may have, such as fear of harming the baby or uncertainty in handling newborns. Ensures the intern assesses the father’s overall involvement in infant care and his understanding of his role in supporting both the baby and mother during this process.*)

### Clinical reasoning

The competent candidate recognises the value of KFC. (*Explaining its importance in maintaining the infant’s warmth, promoting bonding, improving feeding and reducing episodes of apnoea. The candidate should also ensure that the intern recognises the father as being a crucial part of the baby’s care, especially in the context of maternal absence and understand the rationale for engaging fathers in this process.*)

The good candidate additionally anticipates potential challenges and responds with clear, evidence-based reasoning. (*Understand and explain how KFC might affect the father’s emotional bond with the baby, how it can empower him in his caregiving role and the long-term developmental benefits for the infant. The candidate demonstrates an ability to teach the intern to adjust recommendations based on specific concerns or limitations, such as the father’s comfort level with prolonged skin-to-skin contact or mobility during the process.*)

### Explaining and planning

The competent candidate uses clear language to explain to the intern: (*The infant will be warm, feed more easily, bond with him and his father, and episodes of apnoea will be less frequent. The candidate should also teach the intern the indications and contraindications of KFC and outline the key benefits. The candidate names and explains the three components of Kangaroo Mother Care (KMC): skin-to-skin contact, breastfeeding and ongoing support*.)

The good candidate additionally ensures that the intern is actively involved in decision-making, paying particular attention to knowledge-sharing and empowerment. *(The candidate addresses any concerns or questions from the intern and encourages open communication, explaining that he is part of a care team, not just a temporary substitute. The good candidate offers to discuss any further concerns with the intern, recognising her key role, and explains the responsibilities of both parents in the continuity of KMC and KFC.*)

**FIGURE 1:**
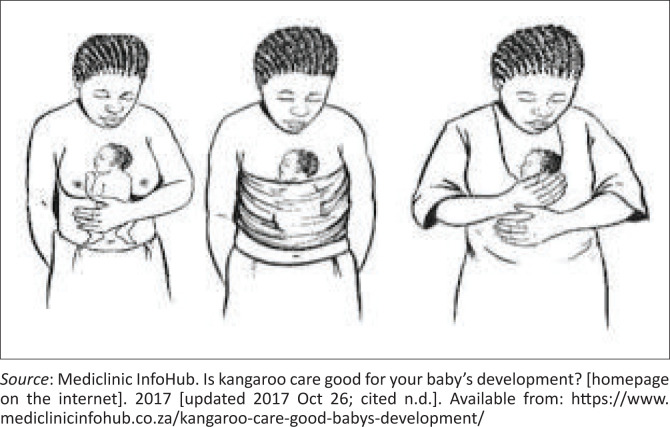
Kangaroo mother care.

**FIGURE 2:**
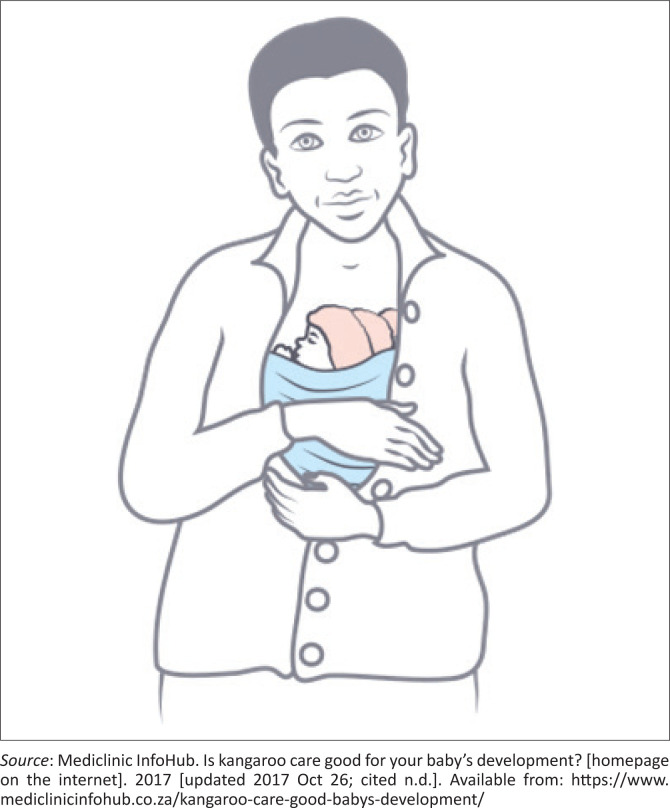
Kangaroo father care.

### Management

The competent candidate proposes appropriate intervention. (*including demonstrating how to properly position the baby skin-to-skin with the father, removing the baby’s clothing for optimal contact, and ensuring the baby is securely tied using a cloth to facilitate KFC. The candidate mentions that the baby should remain in this position for prolonged periods, with brief intervals for monitoring and care.*)

The good candidate demonstrates to the intern how to tie the cloth securely and ensures that the intern acknowledges that the father is comfortable and can move around without the baby falling out. (*They should also discuss the importance of monitoring the baby during KFC, mentioning that the infant’s temperature should be measured twice a day and any signs of distress or illness should be noted. The good candidate teaches the intern that they must teach the father to recognise and respond to danger signs, such as lethargy, changes in feeding, or breathing difficulties, ensuring the infant’s safety during KFC.*) The good candidate allows the intern to reflect on their learning experience and provides appropriate feedback.

## Role player’s instructions

You are a medical intern working in a district-level hospital.

### Opening statement

Doctor, I hear some fathers can help with Kangaroo father care for the babies while the mother takes a break to go home for a while during the day. I have only heard of it, but I have never seen it done before. This father wants to do KFC and has approached me to tell him about it and show him how it is done. Can you tell me more about it and teach me.

### Open responses

Freely tell the doctor:

- *Wants to help but worried you might do something wrong.*
  ■ *Nervous the father might ask him something he doesn’t know*.
  ■ *Have seen KMC before, but unsure if it is the same for fathers*.
  ■ *Not sure how to position the baby securely*.
  ■ *Not sure when KFC is appropriate or if it is going wrong*.

### Closed responses

Only reply this when the candidate asks this specifically:

- Never had hands-on experience with KFC.
- Knows of the benefits of KMC, but not KFC.
- Struggling to explain things clearly and easily understandable to patients.

### Ideas, concerns and expectations

- *Ideas*: Understands that KFC is about skin-to-skin contact between father and baby, but unsure how to teach it a way that makes sense to parents.
- *Concerns*: Worried that you won’t explain things clearly or miss something important.
- *Expectations*: To learn how to do and explain KFC to a father confidently and teach them to do it safely and help his wife. Wants to know when KFC should and should not be done.

### Examination findings

- **The father:**
  ■ Stable.
  ■ Healthy and willing to take on the role of KFC.
  ■ No known allergies or conditions that might interfere with holding the baby.
- **The baby:**
  ■ A term baby weighing about 2.8 kg.
  ■ The baby appears healthy with no signs of distress.
  ■ Stable condition but low weight for age; therefore, KFC is recommended for additional warmth and care.
  ■ Feeding well, with no apnoea episodes reported so far.

**Further reading**

- Mash B, Brits H, Naidoo M, Ras T, editors. South African Family Practice Manual. 4th ed. Pretoria: Van Schaik Publishers, 2023; p. 144–146.
- Dong Q, Steen M, Wepa D, Eden A. Exploratory study of fathers providing Kangaroo Care in a Neonatal Intensive Care Unit. J Clin Nurs. 2022. https://doi.org/10.1111/jocn.16405
- Dipnur QD, Nur B, Candidate M, et al. Exploratory study of fathers providing Kangaroo Care in a Neonatal Intensive Care Unit. J Clin Nurs. 2022:1–12. https://doi.org/10.1111/jocn.16405

